# Twelve tips for final year medical students undertaking clinical assessment

**DOI:** 10.12688/mep.20122.2

**Published:** 2025-04-14

**Authors:** Bunmi S Malau-Aduli, Richard B Hays, Shannon Saad, Karen D'Souza

**Affiliations:** 1 School of Medicine and Public Health, The University of Newcastle, Callaghan, New South Wales, Australia; 2 James Cook University, Townsville, Queensland, Australia; 3 NSW Virtual Hospital, Sydney, New South Wales, Australia; 4 Deakin University, Burwood, Victoria, Australia

**Keywords:** Clinical assessment, OSCE, Decision-making, Professionalism, workplace-based assessment

## Abstract

**Background:**

Clinical assessors in pre-registration examinations have been shown to make decisions about student performance by drawing on two overlapping, yet slightly different perspectives: achieving academic learning outcomes, and contributing to clinical workplace function. The implication for senior medical students is that they should be aware that in ‘final’ clinical assessments they may be judged from both academic and workplace perspectives, where the emphasis may be on how well the candidate would fit into a clinical team, demonstrating reliability, trustworthiness, teachability and ‘safety’.

**Methods:**

This article presents 12 tips for how senior medical students may demonstrate progress towards achieving ‘work readiness’, and so improve performance in assessments close to graduation.

**Results:**

Clinical assessors may include judgment of how well the candidate might work as a junior member of a clinical team, particularly when candidates perform at the borderline level and where assessors are more experienced. This judgment is based on an impression of the student’s demonstration of reliability, trustworthiness, patient safety and teachability. While the underpinning theory was explored in final OSCEs, the suggestions may also be relevant to workplace-based clinical learning and assessment.

**Conclusions:**

Senior medical students should prepare for clinical assessments that will consider how they respond to professionally challenging workplace scenarios as well as essential knowledge and skills.

## Introduction

Clinical assessment remains an essential component of medical education as this is where application of knowledge skills and behaviours can be observed in the context of genuine health care practice. As medical students move towards their senior clinical years, the focus evolves from assessment of foundation knowledge and skills (components of competence) to assessment of how these components are combined as required and applied in clinical practice (competence and performance). While all assessment methods and formats can contribute, observation of clinical performance remains essential, whether by simulation in Objective Structured Clinical Examinations (OSCEs) or more authentically in Workplace-based assessment (WBA) such as during mini-Clinical Evaluation Exercises (mini-CEX); Case-based Discussion (CbD) and Direct Observation of Procedural Skills (DOPS) assessment tasks, among others.

Assessment of medical students in ‘final year’ examinations has two complementary but slightly different roles. The more obvious is to provide evidence that the student has mastered the curriculum at or above the appropriate standard and is eligible for conferral of a university degree ( Epstein, 2007). This comes at the end of years of learning and assessment that should be systematic, comprehensive across curriculum domains and based on data from multiple methods, occasions and judges ( Boursicot *et al.*, 2011; Norcini *et al.*, 2018). The second purpose, perhaps of greater importance to clinicians, the public and employers, is to demonstrate ‘work readiness’, based on the finding that despite meeting academic course requirements, a small number of graduates do not perform satisfactorily as junior doctors in the complex healthcare environment ( Chow *et al.*, 2022; Malau-Aduli *et al.*, 2022a; Monrouxe *et al.*, 2018).

The transition at graduation from primary medical qualification to junior doctor can be a steep learning curve ( Dare *et al.*, 2009; AMC-MBA, 2019). At this level OSCE stations tend to be more complex than for junior students, often including scenarios where combinations of competencies must be utilised to complete tasks to a satisfactory standard ( Burns *et al.*, 2017). Clinicians from the healthcare system are often included as OSCE assessors. Recent research has shown that clinical assessors in pre-registration OSCEs make decisions about student performance by drawing on two overlapping, yet slightly different perspectives ( Malau-Aduli *et al.*, 2021). The first is achievement of program learning outcomes, using the checklists and rating scales provided by the academic institution. The second is how well each candidate might contribute to clinical workplace function. This judgement is aided by using an experience-based heuristic (a cognitive short-cut representation) of a ‘(prototypical) competent first year graduate’ with whom they will soon work. The basis for forming this heuristic is generally drawn from their previous experience of working with new graduates. This heuristic may focus more on professional behaviours (reliability, trustworthiness, teachability) and patient safety and may be more likely to be reflected in a global rating ( Malau-Aduli *et al.*, 2022b).

The implications for senior medical students are that in final assessments by clinical assessors they may be judged more as a new graduate than a senior student. This is more than achieving the academic graduate outcomes as it includes how well the candidate would fit into a clinical team, demonstrating reliability, trustworthiness, teachability and ‘safety’. It is important to note that when mentioning ‘fitting into a clinical team’ – the authors are calling upon students to display behavioural attributes important to safe performance within the team setting (e.g. professionalism, understanding of own role and limitations to scope of practice, respectful communication); and not simply mirroring the attributes of their supervisors.

This article presents 12 tips for how senior medical students may accelerate progress towards achieving ‘work readiness’ and so improve demonstrated performance of crucial traits that clinical assessors are looking for in clinical assessments close to graduation. While the underpinning theory was explored in final OSCEs, the suggestions are likely to be relevant to workplace-based and other clinical assessments. Tips 1, 2 and 3 will assist students prepare for and approach a clinical assessment like an OSCE; tips 4–11 relate to performance during a clinical assessment task; and tip 12 refers to the process of seeking, reflecting on and using feedback and evaluative judgement skills to improve clinical practice.

**Tip 1. Calibrate your performance to that of a newly graduated doctor:** Recognise that examiners might assess you against a personal mental image of the ideal attributes of their own junior medical staff, which should set the standard for what final year health professional students are aiming for. Based on your observation of clinical teams working to provide healthcare - consider what a capable new medical graduate would do in each situation, drawing from your observation and interaction with junior medical staff. It is very important that you prepare for exit-level clinical assessment by participating in learning in the workplace context and actively learn/work alongside junior doctors. Aligning your approach with this prototype can positively influence your assessment, as well as prepare you for entry into clinical practice as a graduate. This ‘calibration’ is part of developing evaluative judgement, that is, the capability to make decisions about the quality of work of oneself and others ( Tai *et al.*, 2018). In the clinical workplace, consistency in performance is important for patient safety and quality care. Aligning your performance with a standard or prototype ensures that patients receive high quality, uniform care regardless of who is providing it ( Malau-Aduli *et al.*, 2021). This contributes to seamless teamwork and continuity of care. Repeating this task of understanding the expected standard, and comparing one's own performance against this standard, develops skills that will be utilised in lifelong learning - commencing within medical school but then exercised during continuing professional development as a clinician.

**Tip 2. Focus on optimising performance:** While adhering to the provided tasks which are closely aligned to the marking criteria, understand that they might not capture all nuances of clinical practice. Strive to optimise your performance, beyond simply meeting the minimum expected standard, to demonstrate the depth and breadth of your understanding and your authentic experience in the clinical learning environment. Showcase your critical thinking, ethical considerations, and holistic approach to patient care. This can assist you score more highly on the marking rubric, as clinical assessment is generally criterion-referenced. Be aware that healthcare settings demand more than just fulfilling the basics. Presenting as a professional demonstrates awareness of your place in the clinical team, the unique context of each individual patient, and the responsibility that team has for those under its care. The best way to ensure that you gain this awareness of how clinical teams work to optimise the health of each individual patient is best gained by active participation in clinical patient care, under the supervision of the team. Clinical assessors often pose the question “will this student be a good fit as a commencing member of my clinical team” when judging clinical performance ( Malau-Aduli *et al.*, 2022b). Demonstrating safe practice, adapted to this patient and clinical setting, showcases your ability to think critically and provide comprehensive care, which is essential for delivering optimal patient outcomes. Striving for excellence aligns with the commitment of healthcare providers and systems to continuous improvement.

**Tip 3. Be prepared for deviations:** Recognise that real-world scenarios might not always perfectly match the scripted situations provided during simulated clinical assessment tasks. Clinical environments are dynamic and unpredictable. Patients' conditions can change rapidly, requiring swift adjustments. Should the status of the OSCE station patient change, be ready to respond effectively. Demonstrating flexibility and adaptability ‘in the moment’ to foster patient safety in unexpected situations is core to healthcare practice and may be viewed positively by clinical assessors ( Chaou *et al.*, 2021). For example, an OSCE station may commence as a history taking station, then become a ‘deteriorating patient station’ if the standardised patient appears to become clinically worse or unresponsive, and the candidate should be prepared to manage this sudden deterioration.

**Tip 4. Emphasise 'What' and 'How':** Remember that the assessment process involves evaluating not just 'what' you do, but also 'how' you approach tasks. Pay attention to the thought processes you demonstrate, as well as your organisational and communication skills. Assessors want to know how you made decisions and may assess your clinical reasoning by following what information you gather and in what sequence. There are often marks for demonstrating purposeful information gathering that informs appropriate clinical reasoning and management ( Gruppen, 2017) – rather than either of a rigid recital of checklists OR appearing haphazard and unstructured in your approach to this particular case. Clinical assessors prefer flexibility and adaptability – tailored to this particular scenario and patient in a particular clinical context. If you go back and forth between history taking and examination, often necessary as potential diagnoses are considered, explain why and consider ‘thinking aloud’ so that others can follow your diagnostic reasoning. In the clinical setting, decisions encompass actions and underlying thought processes, necessitating effective communication of clinical judgment for collaboration, patient education, and informed choices. Therefore, striking a balance between structured analysis and adaptability is essential for evidence-based decisions and patient-centred care.

**Tip 5. Understand your tasks on the station:** The specific tasks outlined in the provided student information for each station are the central point in OSCEs ( Harden & Gleeson, 1979); and are listed to illustrate to learners the steps required to demonstrate competence in each clinical scenario being assessed – for example: take a focused history and clinical examination; and then explain your findings to the patient. Make sure to address all the key points outlined in the tasks as these are closely aligned with the marking criteria; which have been designed to guide the assessor as to what would constitute a comprehensive clinical performance on the selected tasks in the workplace – in order to demonstrate a reliable approach to this particular clinical scenario ( Khan *et al.*, 2013). These tasks outline the key aspects that examiners are looking for in your performance and they serve as a guideline for examiners and provide reassurance that your performance is being assessed objectively. Take time to understand the expectations and focus on addressing each task. This will ensure that you cover all essential requirements of the clinical scenario comprehensively and demonstrate your knowledge and skills (and potentially your dependability as a junior medical team member) effectively.

**Tip 6. Display organisational skills:** Structured and organised responses are crucial. Present your thoughts and actions in an organised, clear, logical manner. This can enhance the clarity of your assessment and showcase your professionalism ( Papadakis *et al.*, 2004). Healthcare settings are fast-paced and demanding. Being organised enhances your efficiency and effectiveness. Accurate record-keeping, time management, and efficient workflows contribute to smoother operations and improved patient care. These skills are generally learned through observing and providing authentic clinical care within healthcare teams and may reflect a learner’s familiarity with the current healthcare provision gleaned from clinical exposure. Clinical assessors value reliability and responsibility when determining that a candidate has demonstrated competence in achieving the learning standard expected for this stage of the course ( Burns *et al.*, 2017; Hendelman & Byszewski, 2014; Papadakis *et al.*, 2004).

**Tip 7. Display a professional demeanour:** Acknowledge that emotions can play a role in assessment judgments. Examiners might consider affective factors such as their own comfort level and confidence in your ability to perform as a junior healthcare professional. Exude confidence, stay composed under pressure, and communicate clearly. Show empathy towards patients and professionalism in your interactions. Demonstrating these qualities can assist you score more highly on the marking rubric. Your confidence and poise can positively impact your overall performance evaluation ( Heyhoe *et al.*, 2016). Be cognisant that within clinical settings, a composed and confident demeanour is vital in patient and team interactions. It reassures patients and instils trust in your abilities. This is crucial for building rapport with patients, collaborating with colleagues, and maintaining a positive healthcare environment. It is also important to recognise that over-confidence will be easily detected by a clinical assessor – remember to remain within the expectations for your level of training/role within the team, and always be honest if you do not know a fact and state that you would seek assistance or look it up.

**Tip 8. Prioritise patient safety:** Examiners place a significant emphasis on patient safety that is demonstrated with logical consideration of each clinical situation. This is a strong driver of clinical practice standards as the focus increases on quality of care ( Dingley *et al.*, 2008). We may not make everyone better, but we must not cause anyone harm. Always consider the safety of the patient and communicate your reasoning in a calm manner, drawing upon your training in graded assertiveness and speaking up to prevent errors or adverse outcomes. Be strict about correct hand hygiene procedures before, between and during clinical encounters. Always keep patient well-being at the forefront of your decisions and actions. If you're unsure about a certain aspect, prioritise safety over other considerations and focus on patient well-being in your response. Students may need to call upon other team members or more senior medical practitioners as part of delivering safe clinical care, at the appropriate level of a senior medical student/junior medical officer. In the clinical setting, errors can have severe consequences. Prioritising patient safety demonstrates your commitment to ethical practice and patient-centred care ( Kwame & Petrucka, 2021). It's a core value that safeguards both patients and the reputation of the healthcare institution – and is highly valued by assessors in determining clinical competence in the workplace.

**Tip 9. Seek clarity if necessary:** If a scenario or task is unclear, don't hesitate to ask for clarification. Never guess or make something up. Asking for clarification is better than making assumptions that might lead to incorrect actions. Clinician assessors value ‘teachable’ junior colleagues and will accept some knowledge gaps so long as these are acknowledged and form part of future professional development ( Hyde *et al.*, 2020). ‘Teachability’ is important for junior doctors and is therefore taken into account during the examination – remember that junior doctors practise under supervision and are still learning while working. Demonstrating a commitment to asking questions to fully understand the situation indicates your dedication to providing accurate and informed responses. Achieving clarity of tasks is essential for the delivery of safe and effective clinical care.

**Tip 10. Demonstrate honesty:** Never tell untruths through either commission or omission. Clinical assessors value honesty and trustworthiness when making judgements about clinical performance ( Malau-Aduli *et al.*, 2021; Malau-Aduli *et al.*, 2022b). For example, if you have forgotten the answer to a question, say so rather than saying ‘we have not covered that yet’ or making something up. You should also state how you will seek the relevant knowledge and skills, e.g. by consulting a more senior team member or by looking up a specific reference source. Clinicians have reasonable knowledge of what you should have learned – and are able to provide greater direction to support you to address any knowledge or skills gaps detected by the assessment once they understand any areas where further training is required. Honesty is an essential component of patient-safety and crucial to building trust within clinical teams, thus forming an essential component of clinical performance assessment ( Kotzee *et al.*, 2017).

**Tip 11. Demonstrate awareness of your scope of practice:** If in doubt, admit what you do not know or feel confident about and explain how you would work out what to do to ensure that safe and effective clinical care is provided. Clinicians are more likely to ‘trust’ a junior colleague who shows insight and awareness of their strengths and weaknesses and knows how to find the right way forward. It is important to be prepared to say ‘could you please help me with this task’ when you do not feel adequately prepared or familiar with assigned clinical tasks. In the workplace, recent graduates are employed under the supervision of more senior team members – they are not expected to practice in isolation. Over-confidence may alarm assessors, whereas ‘entrustability’ is becoming more important ( Weller *et al.*, 2014). A core feature of being trusted by supervisors is demonstrating that more junior learners demonstrate willingness to call for help when required.

**Tip 12. Use each assessment as an opportunity to build your skills in reflective practice:** After each OSCE or clinical assessment, reflect on your performance in order to foster a professional development mindset toward lifelong learning. Analyse both 'what' you did and 'how' you approached the tasks, then consider what you did well and where you could improve. Does your own analysis align with the feedback you were provided? Comparing your self-analysis with input from others and clinical outcomes will develop your skills in reflective practice and evaluative judgment; and discourage unhelpful self-recrimination. Think about how you could improve your approach to align better with the attributes of an effective and safe junior doctor. Identify areas for growth and develop strategies to enhance your future assessments, as well as your clinical practice. Seek, process and accept constructive feedback and act on that feedback ( Boud & Molloy, 2013). Self-reflection is a powerful tool for continuous professional development, which is a universal requirement of ongoing registration as a medical practitioner ( Winkel *et al.*, 2017). Demonstrating reflective practice with a quality improvement mindset provides an approach to lifelong learning, a core attribute of the profession. In the clinical workplace, learning is continuous. Reflecting on your performance allows you to identify strengths and areas for improvement. This proactive approach to self-assessment and the development of evaluative judgment ( Tai *et al.*, 2018) supports ongoing professional development, leading to better patient care and outcomes.

## Conclusion

OSCEs are designed to assess a range of skills and attributes. By understanding the attributes that assessors consider when assessing candidates, medical students can tailor their approach to showcase their abilities effectively and increase their chances of success. Senior medical students should prepare for clinical assessments that will consider more than essential knowledge and skills. Clinical assessors may include judgment of how well the candidate might work as a junior member of a clinical team, particularly when candidates perform at the borderline level and where assessors are more experienced. This judgment is based on an impression of the student’s demonstration of reliability, trustworthiness, patient safety and teachability.

## Disclosure statement

The authors report no conflicts of interest. The authors alone are responsible for the content and writing of the article.

**Practice points**

- Senior medical students should be aware that in ‘final’ clinical assessments they may be judged from both academic and workplace perspectives.
- Clinical assessors may include judgment of how well the candidate might work as a junior member of a clinical team, particularly when candidates perform at the borderline level and where assessors are more experienced.
- Demonstrating reflective practice with a quality improvement mindset and professional role familiarity may enhance the perception of work-readiness by clinical assessors.

## Data Availability

No data are associated with this article.
