# Supplementary material for: Twelve tips for final year medical students undertaking clinical assessment
Source: MedEdPublish (2016). 2025 Apr 14;14:21. Originally published 2024 Apr 15. [Version 2] doi: 10.12688/mep.20122.2 (PMC12082069; doi:10.12688/mep.20122.2)
Supplement: Peer review history

# Reviewer report: Reviewer response for version 2

**DOI:** 10.21956/mep.22505.r41466

**Published:** 2025-05-15

**Authors:** Alexandra M J Langers

**Affiliations:** 1 Leiden University Medical Center, Leiden, The Netherlands

I would like to thank the authors for the revised version of the manuscript. I believe the manuscript has substantially improved by the changes and I have no further comments.

If evidence from practice is presented, are all the underlying source data available to ensure full reproducibility?

Not applicable

Is the topic of the practical tips discussed accurately in the context of the current literature

Yes

Are all factual statements correct and adequately supported by citations?

Yes

Are the conclusions drawn balanced and justified on the basis of the presented arguments?

Yes

Are arguments sufficiently supported by evidence from the published literature and/or the authors’ practice?

Yes

Reviewer Expertise:

Skills acquisition, faculty development, assessment

I confirm that I have read this submission and believe that I have an appropriate level of expertise to confirm that it is of an acceptable scientific standard.

# Reviewer report: Reviewer response for version 2

**DOI:** 10.21956/mep.22505.r41467

**Published:** 2025-04-25

**Authors:** Ayad M Al-Moslih

**Affiliations:** 1 School of Medicine, University of Central Lancashire, Preston, England, UK

Respected authors, 

 Thanks for your elaborate responses and thorough review. The article narrative is evidently improved after clarification of certain terms and elimination of vague ones, and the current adopted sequence of tips is indeed logical.

If evidence from practice is presented, are all the underlying source data available to ensure full reproducibility?

Not applicable

Is the topic of the practical tips discussed accurately in the context of the current literature

Yes

Are all factual statements correct and adequately supported by citations?

Yes

Are the conclusions drawn balanced and justified on the basis of the presented arguments?

Yes

Are arguments sufficiently supported by evidence from the published literature and/or the authors’ practice?

Yes

Reviewer Expertise:

Clinical Skills Assessment, Clinical Training, Preparedness and training.

I confirm that I have read this submission and believe that I have an appropriate level of expertise to confirm that it is of an acceptable scientific standard.

# Reviewer report: Reviewer response for version 1

**DOI:** 10.21956/mep.21547.r37637

**Published:** 2024-07-15

**Authors:** Ayad M Al-Moslih

**Affiliations:** 1 School of Medicine, University of Central Lancashire, Preston, England, UK

Thanks for sharing this article.

 General feedback:

 This article as the title clearly informs us is about Twelve tips for final year medical students undertaking clinical assessment. It is adequately supported by sufficient number of relevant literature citations to support certain propositions. The conclusions drawn are mostly balanced and justified on the basis of the presented arguments. The article, nonetheless, has a potential for further improvement to enhance its suitability to the targeted readers through clarification of certain terms and statements as detailed in the feedback below. Such edits may improve its pragmatic application by readers.

 The content seems to cover a wide scope of tips including things to remember during an exam and other tips relating to preparedness for exams and how to succeed. As a reader I would appreciate the sequence of these tips to be rearranged chronologically if the intention was indeed to cover preparedness before clinical assessment (tips 3, 4, 8), performance during assessment (tips 1, 2, 4, 5, 6, 7, 9, 11, 12) , and tips after an assessment is over (tips 10), while ensuring clarity about the context, applicability and timeline of each tip. As a reader I would be interested in “what should I do?”, “when do I do it?” and “how to do so?”.

 A reader of the introduction would assume that there are only two types of observed clinical assessments which are OSCEs and WBA, despite of the existence of several other methods and formats for out of workplace, observed clinical assessment such as direct observation clinical encounter examination (DOCEE) (Hamdy et al., 2003), Integrated Structured Clinical Examinations (ISCEs) (Mattick et al.,  2008), Directly Observed Procedural Skill (DOPS) (Khanghahi and Azar, 2018), Virtual Clinical Encounter Examination (VICEE) (Hamdy et al., 2021), High-fidelity patient simulator (Boulet et al, 2003) and other formats and approaches for observed simulation-based clinical assessment (Kogan et al., 2009; Ryall et al., 2016).

 I would echo the first reviewer’s remarks with regards to concern 1 that seeks clarification to address inclusivity.

 Specific feedback:

 Tip 1:

 The term reliability i.e. “…potentially your reliability as a junior medical team member” was mentioned while describing the importance of understanding the task required in a station.

 It wasn’t clear to me as a reader what this meant in its current context.

 Tip 3:

 "Consider what a capable new graduate would do in each situation"

 I found this a vague advice for medical students as readers of this tip. How can the learner translate that or apply it?. I would expect more clarification on how a final year medical student can find out, learn or be prepared to demonstrate their competence authentically within the expected performance of their level.

 Tip 4: Focus on optimising performance:

 How is that cognitively possible? How can it be translated practically?

 Statements like “Strive to exhibit the attributes of an ideal graduate” and "Demonstrating ideal competencies showcases your ability to provide comprehensive care". A clarity is needed on what does ideal sound like? What does it entail?

 Tip 6 ends with a wise advice “remember to remain within the expectations for your level of training/role within the team, and always be honest if you do not know a fact and state that you would seek assistance or look it up.”  How does this fit with tip 3 "Consider what a capable new graduate would do in each situation"?

 Perhaps it may help if tip 3 was clearly describing a mindset for preparing for the exam, whereas tip 6 is more about a demonstrated performance during an exam.

 From an application perspective I find that tips 3, 4, 5, 8, and 10 are not simply tips that can be remembered and applied during an exam, however they require a prior preparation and rehearsal to be ready to execute.

If evidence from practice is presented, are all the underlying source data available to ensure full reproducibility?

Not applicable

Is the topic of the practical tips discussed accurately in the context of the current literature

Yes

Are all factual statements correct and adequately supported by citations?

Yes

Are the conclusions drawn balanced and justified on the basis of the presented arguments?

Yes

Are arguments sufficiently supported by evidence from the published literature and/or the authors’ practice?

Yes

Reviewer Expertise:

Clinical Skills Assessment, Clinical Training, Preparedness and training.

I confirm that I have read this submission and believe that I have an appropriate level of expertise to confirm that it is of an acceptable scientific standard, however I have significant reservations, as outlined above.

## References

[1] : Reliability and validity of a simulation-based acute care skills assessment for medical students and residents. *Anesthesiology* .2003;99(6) : 10.1097/00000542-200312000-00007 1270-80 10.1097/00000542-200312000-00007 14639138

[2] : Reliability and validity of the direct observation clinical encounter examination (DOCEE). *Med Educ* .2003;37(3) : 10.1046/j.1365-2923.2003.01438.x 205-12 10.1046/j.1365-2923.2003.01438.x 12603759

[3] : Virtual Clinical Encounter Examination (VICEE): A novel approach for assessing medical students' non-psychomotor clinical competency. *Med Teach* .2021;43(10) : 10.1080/0142159X.2021.1935828 1203-1209 10.1080/0142159X.2021.1935828 34130589

[4] : Direct observation of procedural skills (DOPS) evaluation method: Systematic review of evidence. *Med J Islam Repub Iran* .2018;32: 10.14196/mjiri.32.45 45 10.14196/mjiri.32.45 30159296 PMC6108252

[5] : Tools for direct observation and assessment of clinical skills of medical trainees: a systematic review. *JAMA* .2009;302(12) : 10.1001/jama.2009.1365 1316-26 10.1001/jama.2009.1365 19773567

[6] : Content specificity: is it the full story? Statistical modelling of a clinical skills examination. *Med Educ* .2008;42(6) : 10.1111/j.1365-2923.2008.03020.x 589-99 10.1111/j.1365-2923.2008.03020.x 18482090

[7] : Simulation-based assessments in health professional education: a systematic review. *J Multidiscip Healthc* .2016;9: 10.2147/JMDH.S92695 69-82 10.2147/JMDH.S92695 26955280 PMC4768888

# Response

**Published:** 2025-04-04

**Authors:** Bunmi Malau-Aduli

**Affiliations:** School of Medicine and Public Health, The University of Newcastle, Callaghan, New South Wales, Australia

Please note that the Tips have changed in order.   In the below responses, we will refer to the new tip and reference which number it was previously.  The new order of the Tips is: (previous numbers) 3,4,8,5,1,2,6,7,9,11,12,10.

- The content seems to cover a wide scope of tips including things to remember during an exam and other tips relating to preparedness for exams and how to succeed. As a reader I would appreciate the sequence of these tips to be rearranged chronologically if the intention was indeed to cover preparedness before clinical assessment (tips 3, 4, 8), performance during assessment (tips 1, 2, 4, 5, 6, 7, 9, 11, 12) , and tips after an assessment is over (tips 10), while ensuring clarity about the context, applicability and timeline of each tip. As a reader I would be interested in “what should I do?”, “when do I do it?” and “how to do so?”.

**Author Response: **Sequence of tips reorganised, and content added to introduction: “ *Tips 1, 2 and 3 will assist students prepare for and approach a clinical assessment like an OSCE; tips 4-11 relate to performance during a clinical* *assessment task; and tip 12 refers to the process of seeking, reflecting on and using feedback and evaluative judgement skills to improve clinical practice.”*

- A reader of the introduction would assume that there are only two types of observed clinical assessments which are OSCEs and WBA, despite of the existence of several other methods and formats for out of workplace, observed clinical assessment such as 

Response: Content added to introduction to include a range of commonly used clinical assessments. (underlined below). *“…whether by simulation in Objective Structured Clinical Examinations (OSCEs) or more authentically in Workplace-based assessment (WBA) such as during mini-Clinical Evaluation Exercises (mini-CEX); Case-based Discussion (CbD) and Director Observation of Procedural Skills (DOPS) assessment tasks, among others”*

- I would echo the first reviewer’s remarks with regards to concern that seeks clarification to address inclusivity.

**Author Response: **See response to Reviewer 1

- Tip 1: The term reliability i.e. “…potentially your reliability as a junior medical team member” was mentioned while describing the importance of understanding the task required in a station. It wasn’t clear to me as a reader what this meant in its current context

**Author Response:** Tip 5 (previously Tip 1) : Wording changed from “ *reliability*” to “ *dependability*” for clarity

- Tip 3: Consider what a capable new graduate would do in each situation" I found this a vague advice for medical students as readers of this tip. How can the learner translate that or apply it?. I would expect more clarification on how a final year medical student can find out, learn or be prepared to demonstrate their competence authentically within the expected performance of their level.

**Author Response:** Tip 1 (previously Tip3) Content added to elucidate how learners can establish this level (underlined below). “ *Based on your observation of clinical teams working to provide healthcare* - *consider what a capable new medical graduate would do in each situation, drawing from your observation and interaction with junior medical staff. It is very important that you prepare for exit-level clinical assessment by participating in learning in the workplace context, and actively learn/work alongside junior doctors.* “

- Tip 4: Focus on optimising performance: How is that cognitively possible? How can it be translated practically? Statements like “Strive to exhibit the attributes of an ideal graduate” and "Demonstrating ideal competencies showcases your ability to provide comprehensive care". A clarity is needed on what does ideal sound like? What does it entail?

**Author Response: **Wording changed to remove the word ideal, and focus on optimising performance: Tip 2 (previously Tip 4) “ *exhibit the attributes of an ideal graduate*” changed to “ *optimise your performance*” “ *demonstrating ideal competencies*” changed to “ *demonstrating safe practice, adapted to this patient and clinical setting*”

- Tip 6 ends with a wise advice “remember to remain within the expectations for your level of training/role within the team, and always be honest if you do not know a fact and state that you would seek assistance or look it up.”  How does this fit with tip 3 "Consider what a capable new graduate would do in each situation"?
  Perhaps it may help if tip 3 was clearly describing a mindset for preparing for the exam, whereas tip 6 is more about a demonstrated performance during an exam.

**Author Response: **Tip 3 moved to be Tip 1 (preparation) and Tip 6 moved to be Tip 7 (performance).

# Reviewer report: Reviewer response for version 1

**DOI:** 10.21956/mep.21547.r36655

**Published:** 2024-06-05

**Authors:** Alexandra M J Langers

**Affiliations:** 1 Leiden University Medical Center, Leiden, The Netherlands

This paper provides twelve tips for advanced medical students who have to undertake a clinical assessment exam. The authors refer to the OSCE exam, as this is an often used way to assess students. Based on existing literature, twelve tips are provided that inform students how to be successful in the OSCE exam and make them aware that not only the correct application of knowledge and skills are assessed, but also work readiness and how well the students fit in the team of clinicians is taken into account. The paper provides tips how students can address these themes.  The manuscript is well written. The authors chose to address the OSCE exam and not workplace based assessments, which is a logical choice as it permits to elaborate on tips that are specific for this type of assessment. Literature is well cited and existing literature supports the tips. The information is relevant, as it is important for students to be aware of these implicit aspects that are taken into account during the assessment. I think all tips are relevant, although there is some overlap. Although I have answered in a positive way to the specific questions that were asked in the review format, there are a couple of things I would like to address.

 General remarks:

1. Several times, the authors state that by following the tips, students can demonstrate that they are better than other students. For example, in Tip 4 (This can set you apart from other candidates who might only fulfill the basic requirements) and Tip 6 (Demonstrating these qualities can set you apart from other candidates). I was somewhat surprised by these phrases and wondered why the authors have added them. Is this publication meant for the “happy few” who have access to this paper and are privileged to have extra information, or do the authors want to be inclusive and help all students by creating awareness on how assessment works? If the latter is the case, why mention how to “beat” other students? Please clarify.
2. Often the authors mention that a certain behavior during the exam is preferred, because the assessors like to see this type of behavior. Thereby, I had the impression that the aim of the manuscript is to create test-wise students. If this is the case, I think the authors reach their goal. Personally, I would like to focus more on the relevance for junior doctors. I see that often the connection with the situation of a junior doctor is made, but by first addressing the assessors point of view, I sometimes had the impression while reading the manuscript that some of these tips sound like “secrets how to pass the test” that students tell from one student to another. An example: instead of stating that “clinical assessors prefer teachable junior colleagues” (Tip 9) the authors could elaborate more on why teachability is important and use that argument. So instead of: “the assessor finds this important so you should to that” the authors might frame it like: “teachability is important for junior doctors and is therefore taken into account during the examination”.
3. A third point I would like to mention, but it will not be able to address this in an easy manner, is that this manuscript encourages students to show that they fit in the current system and if there is a good fit, they will get a good pass mark. Over the last couple of years, a lot has been written on topics like diversity and inclusion and, for example, similarity bias. It is important that assessors become aware of this bias and realize that they might (unconsciously) favor students who resemble the current medical staff. The authors might consider to dedicate a short paragraph to this. Now it only focusses on how to make the students fit perfectly in the current system; however, it is also important for the assessors to learn how to appreciate and embrace diversity.

Specific points:

 Tip 1: the introduction suggests that 12 tips will be provided on how to demonstrate work readiness. However, the first tip starts with mentioning the importance of “ticking all the boxes”, and not to forget any of the required tasks, referring to the marking criteria. I was not expecting a tip like this and it took me to the final sentence of the paragraph to understand a potential relation with work readiness. To make the transition between the introduction and the first tip smoother, the authors might consider to first highlight that reliability is an important aspect of work readiness and then continue explaining how this translates to full completion of the tasks.

 Tip 5: to prevent too much overlap, this tip could benefit from a shorter introduction without referring to tip 1 and 2, and focus more on clinical reasoning and making the thoughts of the students explicit.

 Tip 10 and Tip 12: both tips are about self-reflection and there is quite some overlap between these two tips. I would consider merging these two tips and adding one more tip, for example on how to react upon feedback (although this is also addressed in Tip 9). The authors might consider focusing on clarification in case of unclear instructions in Tip 9, and add a new Tip on how to handle feedback (appreciate the feedback as a helpful tool to improve, do not end up in a discussion with the assessor about the feedback provided.

If evidence from practice is presented, are all the underlying source data available to ensure full reproducibility?

Not applicable

Is the topic of the practical tips discussed accurately in the context of the current literature

Yes

Are all factual statements correct and adequately supported by citations?

Yes

Are the conclusions drawn balanced and justified on the basis of the presented arguments?

Yes

Are arguments sufficiently supported by evidence from the published literature and/or the authors’ practice?

Yes

Reviewer Expertise:

Skills acquisition, faculty development, assessment

I confirm that I have read this submission and believe that I have an appropriate level of expertise to confirm that it is of an acceptable scientific standard, however I have significant reservations, as outlined above.

# Response

**Published:** 2025-04-04

**Authors:** Bunmi Malau-Aduli

**Affiliations:** School of Medicine and Public Health, The University of Newcastle, Callaghan, New South Wales, Australia

Please note that the Tips have changed in order.   In the below responses, we will refer to the new tip and reference which number it was previously.  The new order of the Tips is: (previous numbers) 3,4,8,5,1,2,6,7,9,11,12,10.

 - Several times, the authors state that by following the tips, students can demonstrate that they are better than other students. For example, in Tip 4 (This can set you apart from other candidates who might only fulfill the basic requirements) and Tip 6 (Demonstrating these qualities can set you apart from other candidates). I was somewhat surprised by these phrases and wondered why the authors have added them. Is this publication meant for the “happy few” who have access to this paper and are privileged to have extra information, or do the authors want to be inclusive and help all students by creating awareness on how assessment works? If the latter is the case, why mention how to “beat” other students? Please clarify.

**Author response: **Wording changed to reflect the aim for improved performance in the assessment rather than in comparison to peers.

 e.g.

 Tip 2 (previously Tip 4)

 “ *This can set you apart from other candidates who might only fulfill the basic requirements.* “

 Has been changed to :

 “ *This can assist you score more highly on the marking rubric, as clinical assessment is generally criterion-referenced.*  “

 - Often the authors mention that a certain behavior during the exam is preferred, because the assessors like to see this type of behavior. Thereby, I had the impression that the aim of the manuscript is to create test-wise students. If this is the case, I think the authors reach their goal. Personally, I would like to focus more on the relevance for junior doctors. I see that often the connection with the situation of a junior doctor is made, but by first addressing the assessors point of view, I sometimes had the impression while reading the manuscript that some of these tips sound like “secrets how to pass the test” that students tell from one student to another. An example: instead of stating that “clinical assessors prefer teachable junior colleagues” (Tip 9) the authors could elaborate more on why teachability is important and use that argument. So instead of: “the assessor finds this important so you should to that” the authors might frame it like: “teachability is important for junior doctors and is therefore taken into account during the examination”.

**Author response:**Explicit references to the role of a junior doctor have been increased.

 e.g.

 Tip 9 (unchanged number)

 “ *Teachability’ is important for junior doctors and is therefore taken into account during the examination – remember that junior doctors practise under supervision, and are still learning while working*.”

 - A third point I would like to mention, but it will not be able to address this in an easy manner, is that this manuscript encourages students to show that they fit in the current system and if there is a good fit, they will get a good pass mark. Over the last couple of years, a lot has been written on topics like diversity and inclusion and, for example, similarity bias. It is important that assessors become aware of this bias and realize that they might (unconsciously) favor students who resemble the current medical staff. The authors might consider to dedicate a short paragraph to this. Now it only focusses on how to make the students fit perfectly in the current system; however, it is also important for the assessors to learn how to appreciate and embrace diversity.

**Author response:**New content added to define what “fitting in” does and does not mean:

 Added to the introduction:

 “ *It is important to note that when mentioning ‘fitting into a clinical team’ – the authors are calling upon students to display behavioural attributes important to safe performance within the team setting (e.g. professionalism, understanding of own role and limitations to scope of practice, respectful communication); and not simply mirroring the attributes of their supervisors*.”

 - Tip 1: the introduction suggests that 12 tips will be provided on how to demonstrate work readiness. However, the first tip starts with mentioning the importance of “ticking all the boxes”, and not to forget any of the required tasks, referring to the marking criteria. I was not expecting a tip like this and it took me to the final sentence of the paragraph to understand a potential relation with work readiness. To make the transition between the introduction and the first tip smoother, the authors might consider to first highlight that reliability is an important aspect of work readiness and then continue explaining how this translates to full completion of the tasks.

**Author response:**Additional content added to Tip 1 to clarify the workplace relevance of the marking criteria (underlined below)

 e.g.

 Tip 5 (previously Tip 1)

 “ *Make sure to address all the key points outlined in the tasks as these are closely aligned with the marking criteria; which have been designed to guide the assessor as to what would constitute a comprehensive clinical performance on the selected tasks in the workplace – in order to demonstrate a reliable approach to this particular clinical scenario* ”

 - Tip 5: to prevent too much overlap, this tip could benefit from a shorter introduction without referring to tip 1 and 2, and focus more on clinical reasoning and making the thoughts of the students explicit.

**Author response:**References to the content from Tip 1 and 2 removed, and additional content added to highlight the importance of demonstrating clinical reasoning. (underlined below)

 Tip 4 (previously Tip5) new wording:

 “ *Assessors want to know how you made decisions and may assess your clinical reasoning by following what information you gather and in what sequence. There are often marks for demonstrating purposeful information gathering that informs appropriate clinical reasoning and management (* *Gruppen, 2017* *) – rather than either of a rigid recital of checklists OR appearing haphazard and unstructured in your approach to this particular case.”*

 - Tip 10 and Tip 12: both tips are about self-reflection and there is quite some overlap between these two tips. I would consider merging these two tips and adding one more tip, for example on how to react upon feedback (although this is also addressed in Tip 9). The authors might consider focusing on clarification in case of unclear instructions in Tip 9, and add a new Tip on how to handle feedback (appreciate the feedback as a helpful tool to improve, do not end up in a discussion with the assessor about the feedback provided.

**Author response:**Tip 10 and 12 clarified to highlight their uniqueness.

 Tip 12 (previously Tip 10) : title changed from “ *Practice self-reflection*” to “ *Use each assessment as an opportunity to build reflective practice*”

- Emphasis added on developing a lifelong learning mindset.

Tip  11 (previously Tip 12): title changed from “ *Demonstrate self-awareness of limitations*” to  “ *Demonstrate awareness of your scope of practice*”

- Emphasis added on insight and willingness to request supervisor input.
